# Psychometric properties of the Chinese version of the Trait Gratitude to Nature Scale

**DOI:** 10.3389/fpsyg.2023.1231962

**Published:** 2023-09-27

**Authors:** Xiaoyu Li, Hongyu Liang, Tonglin Jin, Jing Zhang, Yunna A, Mulan Hu, Yifan Wang

**Affiliations:** School of Psychology, Inner Mongolia Normal University, Hohhot, China

**Keywords:** gratitude to nature, scale, Chinese university student, validity, reliability

## Abstract

**Objective:**

The purpose of this study was to evaluate the reliability and validity of the Chinese version of the Trait Gratitude to Nature Scale (TGNS) for Chinese college students.

**Methods:**

The original English version of the TGNS was translated into Chinese. Subsequently, two samples consisting of 1,131 Chinese university students from Inner Mongolia Autonomous Region was recruited through online surveys to evaluate the psychometric properties of the Chinese version of the TGNS, including the discrimination, construct validity, criterion validity, reliability and gender invariance.

**Results:**

The Chinese version of the TGNS showed good psychometric properties. The item-total correlation coefficients of the scale ranged from 0.813 to 0.909. Exploratory factor analysis using data from Sample 1 (*n* = 617) demonstrated that the Chinese version of the TGNS has one factor. The confirmatory factor analysis using data from Sample 2 (*n* = 514) showed that the Chinese version of the TGNS has appropriate construct validity (χ^2^*/df* = 4.157, RMSEA = 0.078, TLI = 0.943 and CFI = 0.967). The significant correlation between the Chinese version of the TGNS and all the other criterion scale scores (*p* < 0.001) indicated that the Chinese version of the TGNS displays good criterion validity. The test–retest reliability was 0.914, using the sub-sample of Sample 2 (*n* = 127). The results of gender invariance test indicated that the Chinese version of the TGNS has entire equivalence between the two genders.

**Conclusion:**

The Chinese version of the TGNS has satisfactory psychometric properties in the Chinese cultural context and can be used as s a reliable and valid instrument to assess trait gratitude to nature.

## 1. Introduction

Nature has given birth to all life and provided numerous benefits to humans. The Millennium Ecosystem Assessment also proposed that nature’s benefits can be categorized into four types of ecosystem services (Reid et al., 2005): provisioning benefits (e.g., air and food), regulating benefits (e.g., water purification), cultural benefits (e.g., spiritual enrichment), and supporting benefits (e.g., soil formation). Thus, expressing gratitude to nature is a consensus of ecological ethics among human beings throughout history, as we are all beneficiaries of nature’s generosity (Lewis, 2023).

### 1.1. The concept and theory of gratitude to nature

Gratitude toward nature arises from recognizing the benefits that nature provides (Kearns, 2020; Manley et al., 2022; Tam, 2022). Gratitude to nature can be expressed as either state or trait gratitude. State gratitude to nature is the episodic experience of gratitude toward nature (Naito et al., 2010; Tam, 2022), while trait gratitude to nature refers to a habitual tendency or emotional trait of feeling gratitude toward nature (Liang et al., 2015; Tam, 2022). Individuals with high trait gratitude to nature could more easily recognize the small benefits which can be attributed to nature and often perceive nature as a benefactor that plays a significant role in their lives (Watkins et al., 2003).

Gratitude to nature is a form of gratitude for non-human objects. As mentioned earlier, state gratitude to nature is usually triggered by nature-related experiences. Several studies have indicated that comprehending the scarcity of natural resources and recognizing their significance to human life can readily contribute to individuals experiencing a state of gratitude to nature (Kearns, 2020; Tam, 2022). In line with the social cognitive model of trait and state levels of gratitude proposed by Wood et al. (2008), the development of gratitude at the trait level is continuing on the habitual accumulation of gratitude at the state level. Thus, trait gratitude toward nature could be cultivated through the continuous accumulation of gratitude for such experiences, eventually leading to the formation of a habitual tendency.

The amplifying the good theory of gratitude proposes that gratitude typically functions to amplify individuals’ appreciation of the various benefits in life (Watkins, 2013). Algoe (2012) further suggests that interpersonal gratitude helps individuals recognize and acknowledge the goodwill of others in their interpersonal relationships, thereby strengthening and maintaining those connections. Expanding on these theories, Tam (2022) argues that while gratitude to nature may not have human benefactor, it can still engender reciprocal behavioral effects, specifically in promoting pro-environmental behaviors. This is because gratitude to nature involves concrete objects that can be reciprocated, which distinguishes it from the more generalized trait or state gratitude.

### 1.2. The function of gratitude to nature

Previous studies have shown that gratitude, as a transpersonal positive emotion, can motivate individuals to take a long-term development vision in economic decisions or tasks that require self-control, such as intertemporal decision-making and public goods games (DeSteno et al., 2014; Kates and DeSteno, 2021). Grateful individuals would delay immediate gain, promote sustainability and reduce the over-consumption of public resources (DeSteno et al., 2014; Kates and DeSteno, 2021). Recent studies have indicated that participants’ gratitude to nature can expand the function of gratitude for the sustainable use of economic resources to the function of preserving natural resources (Naito et al., 2010; Zelenski and Desrochers, 2021; Jacobs, 2022; Tam, 2022). Kearns’s studies indicated that participants who expressed gratitude to nature in the experimental group showed a higher willingness to conserve water compared to the control group, which only read the definition of water (Kearns, 2020). Additionally, other research has observed that participants who wrote gratitude letters to nature displayed greater intentions to engage in pro-environmental behaviors such as recycling, using reusable shopping bags, and utilizing energy-efficient products, relative to the control group (Jacobs, 2022). It is noteworthy that research has demonstrated two key characteristics of the positive predictive effect of gratitude to nature on pro-environmental behavior: Firstly, this predictive effect was not observed when considering interpersonal gratitude (Tam, 2022); Secondly, even after controlling for general trait gratitude, this prediction remained significant (Kearns, 2020).

Additionally, gratitude to nature is believed have a positive impact on individual mental health. Multiple studies have demonstrated that individuals with higher levels of trait gratitude experience lower levels of depression and anxiety, as well as an increased sense of peace of mind and happiness (Ruini and Vescovelli, 2013; Park et al., 2019; Liang et al., 2020). Theoretically, both the amplifying the good theory and the find, remind, and bind theory of gratitude indicate that regardless of whether the object is a thing or a person, the fundamental function of gratitude is to raise individuals’ awareness of the unrecognized benefits in their surroundings (Algoe, 2012; Watkins, 2013; Liang, 2022). This awareness helps individuals move beyond self-centered thinking and reduce feelings of depression, which is commonly referred to as the cognitive focus shift hypothesis of gratitude (Watkins, 2013; Liang, 2022). Building upon this theoretical foundation, it can be posited that trait gratitude to nature should also exhibit a negative correlation with depression. Recent empirical research has further found that that the ability to experience awe and gratitude to nature contributes to an individual’s well-being index, which can serve as an indicator of their mental health status or risk of depression (Büssing et al., 2021).

### 1.3. The measurement of trait gratitude to nature

Most previous studies on the function of gratitude to nature have only discussed its mechanism from the perspective of state gratitude to nature, rather than trait gratitude to nature. This could be attributed to the absence of a reliable and valid scale that can measure gratitude to nature consistently over an extended period (Mitchell, 2012; Tam, 2022). While initially, the “appreciation for simple pleasures” dimension from the Gratitude Resentment and Appreciation Test (GRAT) developed by Watkins and his colleagues (Watkins et al., 2003), could potentially be used to measure gratitude to nature, it also encompasses appreciation for other simple pleasures in life and therefore may not solely focus on gratitude toward nature. Naito later developed the Environmental Attitudes Scale (Naito et al., 2010), which did not differentiate between trait gratitude and state gratitude, nor did it explicitly consider the conceptual network of gratitude to nature (Tam, 2022). Moreover, the Adolescent Gratitude Scale developed by Chinese researchers includes a dimension for measuring gratitude toward nature (He et al., 2012), but its applicability is limited to teenagers, which may restrict the scale’s generalizability to other age groups.

More recently, Tam (2022) developed the Trait Gratitude to Nature Scale (TGNS), drawing inspiration from the Gratitude Questionnaire-6 (GQ-6) developed by McCullough et al. (2002). Originally, it was hypothesized that the TGNS would consist of four distinct dimensions. However, both exploratory factor analysis (EFA) and confirmatory factor analysis (CFA) conducted by Tam (2022) demonstrated that the TGNS is, in fact, unidimensional, aligning with the structure of the GQ-6. Furthermore, the TGNS exhibited a high alpha coefficient of at least 0.84, indicating strong internal consistency (Tam, 2022). Additionally, the analysis of the TGNS has indicated a significant positive correlation between gratitude to nature and pro-environmental behavior (*r* = 0.22 ~ 0.55). Overall, the Trait Gratitude to Nature Scale (TGNS) has demonstrated good reliability and validity across diverse age groups, as noted by Tam (2022) in his studies.

### 1.4. The purpose and significance of this study

The primary objective of this study is to translate and validate the TGNS for utilization within the Chinese cultural context, thereby facilitating gratitude-related research in China. This endeavor holds both theoretical and practical significance.

From a theoretical perspective, by establishing the reliability and validity of the TGNS in Chinese mainland, this study contributes to the extension of the scale’s cross-cultural applicability. Furthermore, by verifying the extent of an individual’s gratitude toward nature, this research endeavors to illuminate the emotional bond that is formed between an individual and the natural environment. Neglecting the emotional dimensions of human-nature relationships, including gratitude to nature, could obscure potential avenues for fostering engagement and devising strategies for environmental conservation (Phillips et al., 2023).

In practical terms, investigating the association between gratitude to nature and pro-environmental behaviors can provide valuable insights for educators seeking to encourage environmental-friendly actions through gratitude interventions. Similarly, exploring the relationship between gratitude to nature and depression can expand the scope of intervention methods employed by gratitude researchers in addressing depressive symptoms, thereby opening up new pathways for gratitude-based interventions in the realm of depression.

## 2. Materials and methods

### 2.1. Participants

We employed a two-wave convenient sampling method to distribute questionnaires to a total of 1,131 valid participants, combining Sample 1 and Sample 2 from three universities in the Inner Mongolia Autonomous Region of China. During the initial distribution of questionnaires, we selected students from a normal university, which naturally led to an uneven gender ratio with more female students and fewer male students. To address this issue and ensure a balanced gender ratio that aligns with the necessary requirements for data analysis (e.g., EFA, CFA, etc.), we extended the sampling range to encompass three different types of universities.

To create an online questionnaire, we utilized the WJX platform, which offers functionalities similar to Qualtrics. The generated questionnaire link on the WJX platform was then shared with groups of university students through social media platforms such as WeChat. To ensure the overall data quality, we also utilized the quality screening function provided by the WJX platform to eliminate any invalid questionnaires. Prior to starting the study, we obtained informed consent from all participants, ensuring their voluntary participation and respecting their right to withdraw. Participants were eligible to receive monetary rewards through the “red packet money” setting function of the WJX platform. These rewards were granted to participants who diligently completed the questionnaire in accordance with the provided instructions. In order to collect tracking data, we adopted the method of student ID matching. This involved utilizing the same student ID to extract matching data. We did not inquire about the significance of each student ID or the corresponding real names, nor do we possess such authority. By using this method, we aim to maximize data anonymity and ensure the privacy of participants as much as possible.

Sample 1 (used for item analysis and exploratory factor analysis): Of the 645 selected, 617 participants completed the scale, resulting in an effective response rate of 95.65%. There were 95 freshmen (15.39%), 144 sophomores (23.35%), 217 juniors (35.17%), and 161 seniors (26.09%). The sample included 299 males and 318 females, with a mean age of 20.39 years (SD = 2.49 years).

Sample 2 (used for confirmatory factor analysis, criterion validity, and test–retest reliability assessment): Out of the 530 selected, 514 participants completed the scale, resulting in an effective response rate of 96.9%. There were 54 freshmen (10.51%), 160 sophomores (31.13%), 151 juniors (29.38%), and 149 seniors (28.98%). The sample included 243 males and 271 females, with a mean age of 21.11 years (SD = 1.62 years). To obtain a test–retest sample, we launched a recruitment announcement for a four-week follow-up study within Sample 2. A total of 135 participants registered to take part in the second measurement. After applying the quality screening function of WJX, we identified 127 sets of matching data. This subset comprised 44 males and 83 females, with a mean age of 19.98 years (SD = 1.17 years). Among them, there were 24 freshmen (18.89%), 51 sophomores (40.17%), 25 juniors (19.68%), and 27 seniors (21.26%).

### 2.2. Measures

#### 2.2.1. Trait Gratitude to Nature Scale

The Trait Gratitude to Nature Scale (TGNS) measures an individual’s trait gratitude toward nature. It comprises 8 items rated on a 7-point Likert scale (1 = strongly disagree to 7 = strongly agree). The scale includes statements such as “I want to express gratitude to nature for its support in my life” and “It is important for me to demonstrate thankfulness to the natural world for the resources I utilize.” A higher score on the TGNS indicates a stronger inclination toward expressing gratitude to nature (Tam, 2022).

To conduct the study, we first secured authorization from the original author of TGNS. Next, we recruited two English-proficient postgraduate students to translate the TGNS from English into Chinese. Subsequently, we employed a back-translation method to assess the translation’s accuracy. Finally, two psychologists specializing in related fields reviewed all the translated versions to ensure both cultural appropriateness and accuracy, ultimately reaching a consensus for producing the preliminary Chinese version of the TGNS.

#### 2.2.2. Gratitude Questionnaire-6

The Gratitude Questionnaire-6 (GQ-6) is a 6-item scale developed by McCullough et al. (2002) to measure trait gratitude on a 7-point Likert scale ranging from 1 (strongly disagree) to 7 (strongly agree). The scale includes two reverse-coded items. Sample statements from the GQ-6 include “I feel that as I grow up, I become more and more grateful for the people, things and situations in my life” and “There is so much in my life that I should be grateful for.” A higher score on the GQ-6 indicates a higher level of trait gratitude. The Chinese version of the GQ-6 was validated by Li et al. (2012), and in the present study, it demonstrated satisfactory reliability with a Cronbach’s α of 0.863.

#### 2.2.3. Pro-environmental Behavior Scale

The Pro-environmental Behavior Scale (PEBS) employed in this study was developed by Liu and Wu (2013). It consists of 11 items that measure pro-environmental behavior using a 5-point Likert scale (1 = never to 5 = always). The scale includes statements such as “I will recycle plastic bags” and “I will bring shopping bags when shopping.” Higher scores on the PEBS indicate a higher level of pro-environmental behavior. Previous research conducted among Chinese college students by Liu and Wu (2013), Li et al. (2019, 2022), and Wang and Huo (2022) has accumulated evidence supporting the scale’s satisfactory statistical indicators. In the current study, the Cronbach’s α coefficient was found to be 0.868.

#### 2.2.4. Center for Epidemiological Studies Depression Scale

The Center for Epidemiological Studies Depression Scale (CES-D), developed by Radloff (1977), contains 20 items for assessing the level of depression. It uses a 4-point scale (0 = less than 1 day, 1 = 1–2 days, 2 = 3–4 days, 3 = 5–7 days) and includes two items that are coded inversely. The scale includes statements such as “I felt lonely.” and “I thought people are unfriendly to me.” A higher score on the CES-D indicates a higher severity of depression. The Chinese version of the CES-D has been shown to possess acceptable psychometric properties and good discriminative validity (Zhang et al., 2010). The Cronbach’s α coefficient in this study was 0.834.

### 2.3. Data analysis procedure

To evaluate the psychometric properties of the initial Chinese version of the TGNS, we conducted item analysis, exploratory factor analysis (EFA), confirmatory factor analysis (CFA), test–retest reliability assessment and gender invariance test.

Sample 1 was utilized to perform item analysis, which aimed to evaluate he discrimination of items and remove those that do not meet the criteria. The initial step in the analysis involves assessing item discrimination using the Extreme Group Method. This method involves comparing the critical ratio (CR), which represents the t-value. Based on relevant recommendations (Cureton, 1957; Kline, 2005), the scores of participants on the Chinese version of the TGNS were ranked, with the top 27% categorized as the high group and the bottom 27% categorized as the low group. Independent samples t-tests were then conducted to compare these groups on each item. It is important to note that a CR (t-value) greater than 3.00 indicate good differentiation between items (Yang et al., 2021). In the second step of the analysis, the homogeneity of the scale items was assessed by examining the corrected item-total correlations. Items with item-total correlations below 0.40 should be considered for deletion (Wu, 2010). The internal consistency was also evaluated using Cronbach’s α value. Items would be retained if deletion did not increase Cronbach’s α coefficient; items would be removed if deletion increased the coefficient.

To examine the latent factor structure of the Chinese version of the Trait Gratitude to Nature Scale (TGNS), exploratory factor analysis (EFA) was conducted in Sample 1 according to established guidelines. Two specific criteria were considered: (1) the Kaiser-Mayer-Olkin (KMO) test value should exceed 0.70, and (2) the significance of Bartlett’s sphericity test should be below the threshold of *p* < 0.001 (Montoya and Edwards, 2021). In addition, we employed parallel analysis to identify the relevant factors. Parallel analysis is considered more stringent in determining the number of factors to extract from the analysis. It recommends retaining factors with eigenvalues higher than the randomly generated eigenvalues of their corresponding components in a simulated dataset, which possesses an equivalent number of items and respondents (Yazdani and Bonsaksen, 2019).

The construct validity of the Chinese version of the TGNS was further evaluated with confirmatory factor analysis on Sample 2. The maximum likelihood estimation method was employed in conducting CFA. The model was considered to be acceptable if the goodness-of-fit index (GFI) > 0.90, normed fit index (NFI) > 0.90, Tucker-Lewis index (TLI) > 0.90, comparative fit index (CFI) > 0.90, incremental fit indexes (IFI) > 0.90, root mean square error of approximation (RMSEA) < 0.08, and the ratio of the chi-square test statistic to degrees of freedom (*χ*^2^/*df*) ≤ 5 (Schneider et al., 2022; He et al., 2023).

**Figure 1:**
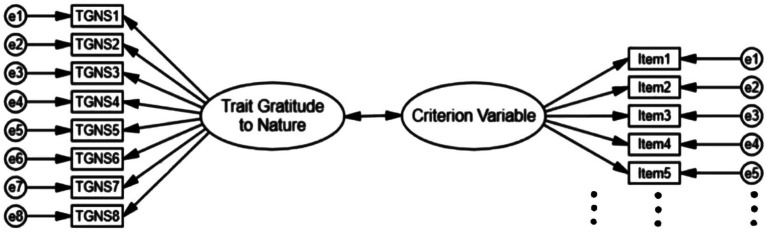
The correlation model for the trait gratitude to nature and criterion variables (*n* = 514).

The criterion validity assessment was evaluated for the Chinese version of the TGNS using data from Sample 2. To improve the precision in accounting for measurement error, we used a SEM approach to estimate associations between latent factors. As shown in Figure 1, we employed a correlation model to evaluate the criterion validity. This model included two measurement models: one for assessing gratitude to nature and another for measuring one of the three criterion variables (such as trait gratitude, pro-environmental behavior, and depression). The correlation between trait gratitude to nature and the respective criterion variable was then estimated.

To assess the test–retest reliability, we used Pearson product–moment correlation analysis to evaluate the consistency of measurements over time (Lin et al., 2019).

To investigate the gender invariance, we employed the common step-wise model comparison approach introduced by Meredith (1993). This approach involves comparing progressively restrictive models, ranging from the unconstrained configural model to the latent mean model, in order to establish increasing levels of invariance (Hong et al., 2003; Schmalbach et al., 2023). In the initial step, we conducted a test of configural invariance by employing Model 1 (configural invariance). In this test, multiple parameters were estimated freely. The purpose of this test was to examine whether the number of latent variables and the relationships between observed variables and latent variables exhibited consistency across gender groups. The results of this test provided the foundation for subsequent analyses and model comparisons. In the second step, we proceeded with Model 2 (weak invariance) to assess the equivalence of factor loadings across different gender groups. Following that, in the third step, we employed Model 3 (strong invariance) to examine the invariance of intercepts of observed variables across the gender groups. Moving on to the fourth step, we utilized Model 4 (strict invariance) to evaluate whether error variances were equivalent across gender groups. Finally, assuming the establishment of Model 4, we proceeded to Model 5 (latent mean invariance) to investigate the invariance of latent variable means across gender groups. It is crucial to note that each subsequent model is nested within the previous one, and the analysis proceeds only if the preceding model holds. If a model fails to meet the criteria for invariance, the testing process is halted at that point. To assess measurement invariance, we examined changes in goodness-of-fit measures, specifically focusing on the RMSEA and CFI. It is considered acceptable for the change in the goodness-of-fit measures to not exceed 0.015 for ΔRMSEA and 0.01 for ΔCFI (Galenkamp et al., 2017).

In the process of data analysis, we employed SPSS 26.0 for conducting exploratory factor analysis and assessing the internal consistency, test–retest reliability of the Chinese version of the TGNS. Additionally, we utilized Amos 21.0 and Mplus 8.0 to perform confirmatory factor analysis, examine criterion-related validity and test for gender invariance.

## 3. Results

### 3.1. Item analysis

Table 1 presents the descriptive statistics, including the mean, standard deviation, the critical ratio, Cronbach’s α (if an item was deleted), and corrected item-total correlations for the eight items of the Chinese version of the TGNS based on the data from Sample 1 (*n* = 617). The critical ratio (CR) of each item in the questionnaire ranged from 20.040 to 29.242, all of which were > 3 (*p* < 0.001). The score of each item was positively correlated with the total score (*r* = 0.813–0.909, *p* < 0.01). After deleting each item individually, Cronbach’s α coefficient of the scale was 0.936–0.945, which did not exceed the original Cronbach’s α coefficient of 0.947. Therefore, all items were reserved.

**Table 1:** Descriptive statistics for items of the Chinese version of the Trait Gratitude to Nature Scale (TGNS, *n* = 617).

Item	Mean	*SD*	Critical ratio	Cronbach’s α, if item deleted	Corrected item-total correlations
I want to give thanks to nature for its support to my life. (我要感谢大自然对我生命的馈赠。)	5.869	1.499	20.040***	0.945	0.813
It is important for me to do something to show my thankfulness to the natural world for the resources I can use. (因为我可以使用自然界的资源, 所以做一些事情来表达我对大自然的感激是很重要的。)	5.954	1.344	21.688***	0.941	0.847
I am deeply grateful to nature for all it provides me with. (我深深感激大自然给我的一切。)	5.951	1.335	24.807***	0.937	0.895
I have a strong sense of gratitude toward the natural world for its support to me. (我非常感激大自然对我的支持。)	5.910	1.303	28.297***	0.936	0.909
If I were to list the reasons why I feel grateful toward nature, it would be a very long list. (如果要列出为什么我对大自然心存感激的原因, 那将是一个非常长的清单。)	5.636	1.479	29.242***	0.939	0.873
As I look back on my life, I feel that I have been richly blessed by the natural world. (回想在我的生命中, 我感受到了自然丰富的恩惠。)	5.914	1.310	23.185***	0.939	0.869
Even something as simple as seeing a beautiful flower can make me feel grateful toward nature. (即使是像是看到一朵美丽的花这样简单的事情也能让我对大自然心存感激。)	5.737	1.433	24.542***	0.943	0.827
Whenever I eat something delicious, I feel grateful toward the natural world for its provisions. (每当我吃到美味的东西, 我都会对大自然的供给心怀感激。)	5.611	1.507	26.760***	0.943	0.828
Total TGNS	5.823			0.947	

***p < 0.001.

### 3.2. Exploratory factor analysis

**Figure 2:**
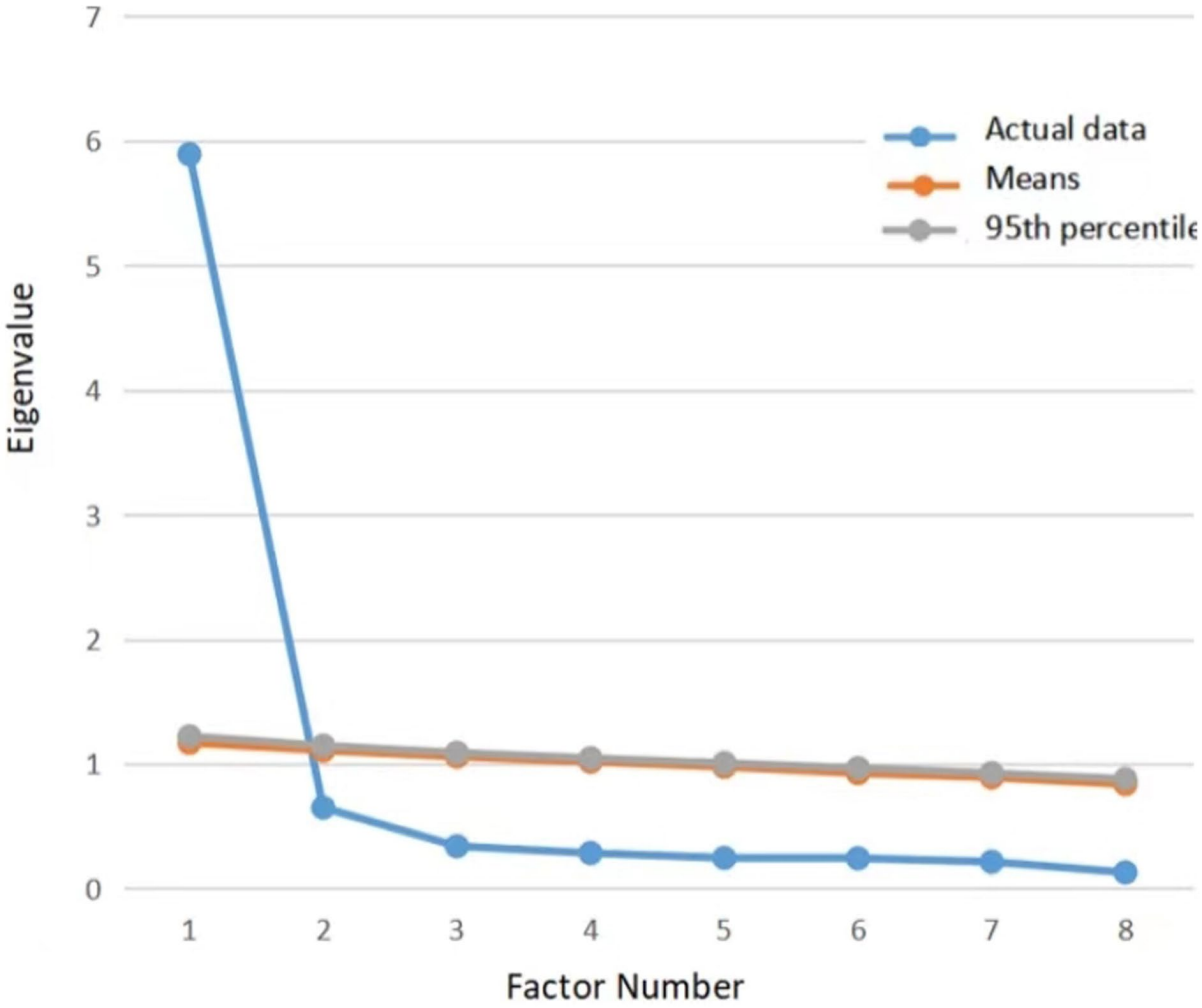
Scree plot of parallel analysis for the Chinese version of the Trait Gratitude to Nature Scale (TGNS, *n* = 617).

Kaiser-Meyer-Olkin (KMO) value of 0.934, and the result of Bartlett’s sphericity test was significant (χ^2^ = 4529.851, *p* < 0.001), suggesting that the correlation matrix was suited for exploratory factor analysis. A single factor was identified with an eigenvalue of 5.894, which accounted for 73.68% of the total variance. The factor loading results for Sample 1 were presented in Table 2. The factor loadings ranged from 0.655 to 0.836, indicating that a strong association between all items and the factor. The results of parallel analysis provided support for the one-factor model, as the estimated eigenvalue for factor 1 (5.894) exceeded the corresponding random estimate obtained from parallel analysis (1.169, 1.221), as depicted in Figure 2.

**Table 2:** Factor loading of the Chinese version of the Trait Gratitude to Nature Scale (TGNS, *n* = 617).

Item	Factor loading
1. I want to give thanks to nature for its support to my life.	0.655
2. It is important for me to do something to show my thankfulness to the natural world for the resources I can use.	0.724
3. I am deeply grateful to nature for all it provides me with.	0.812
4. I have a strong sense of gratitude toward the natural world for its support to me.	0.836
5. If I were to list the reasons why I feel grateful toward nature, it would be a very long list.	0.759
6. As I look back on my life, I feel that I have been richly blessed by the natural world.	0.763
7. Even something as simple as seeing a beautiful flower can make me feel grateful toward nature.	0.675
8. Whenever I eat something delicious, I feel grateful toward the natural world for its provisions.	0.671

### 3.3. Confirmatory factor analysis

**Figure 3:**
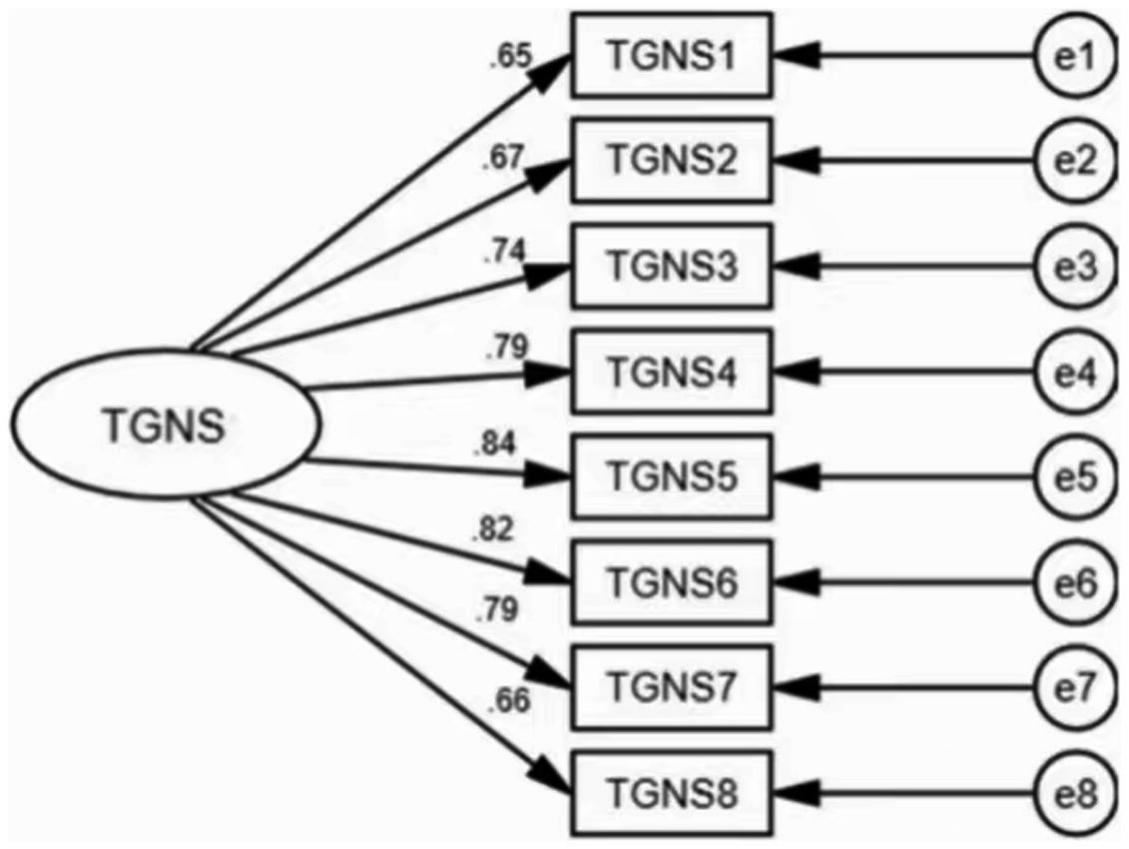
Standardized one -factor model for the Chinese version of the Trait Gratitude to Nature Scale (TGNS, *n* = 514).

In the confirmatory factor analysis results, the model fit for the structure of the Chinese version of the TGNS on the data of Sample 2 was as follows: χ^2^*/df* = 4.157 (*p* < 0.001), RMSEA = 0.078, GFI = 0.969, NFI = 0.974, TLI = 0.943, and CFI = 0.967, indicating that the model had appropriate construct validity. Figure 3 depicted the standardized coefficients of the one-factor model of the Chinese version of the TGNS.

### 3.4. Criterion validity assessment

Table 3 demonstrates that there was a statistically significant correlation between trait gratitude to nature and each of the criterion variables. Specifically, trait gratitude to nature exhibited significant and moderate relationships with trait gratitude (*r* = 0.423, *p* < 0.01), pro-environmental behavior (*r* = 0.329, *p* < 0.01), and depression (*r* = −0.465, *p* < 0.01). In conclusion, the TGNS demonstrated satisfactory criterion validity.

**Table 3:** Fit indices for the correlation models.

Model	χ^2^/df	IFI	CFI	TLI	RMSEA	*r*
Trait gratitude to nature and depression	2.462	0.911	0.911	0.901	0.053	−0.465**
Trait gratitude to nature and trait gratitude	4.099	0.941	0.976	0.962	0.075	0.423**
Trait gratitude to nature and pro-environmental behavior	3.745	0.916	0.916	0.902	0.073	0.329**

*n* = 514. **p < 0.01. IFI, incremental fit indexes; CFI, comparative fit index; TLI, Tucker-Lewis index; RMSEA, root mean square error of approximation; r, the correlation between the trait gratitude to nature and the criterion variable.

### 3.5. Test–retest reliability assessment

The test–retest reliability of the Chinese version of the TGNS was assessed using 127 pairs of valid data, obtained from a re-recruited sample of participants from Sample 2, measured 4 weeks apart. The analysis revealed a test–retest reliability coefficient of 0.914, indicating excellent internal consistency and stability over time.

### 3.6. Gender invariance test

Based on the results presented in Table 4, we can conclude that all five models—M1 (Configural invariance), M2 (Metric invariance), M3 (Scalar invariance), M4 (Strict invariance), and M5 (Latent mean invariance)—demonstrate satisfactory outcomes. These findings suggest that the significance and potential structure of the Chinese version of the TGNS are the same in both male and female gender groups.

**Table 4:** Fit indices for the analysis of gender invariance models (*n* = 514).

Model	χ^2^/df	CFI	△CFI	RMSEA	△RMSEA
M1 (Configural invariance)	4.157	0.967		0.078	
M2 (Metric invariance)	3.118	0.967	0	0.091	0.013
M3 (Scalar invariance)	2.819	0.966	0.001	0.084	0.007
M4 (Strict invariance)	3.105	0.957	0.009	0.091	0.007
M5 (Latent mean invariance)	2.995	0.955	0.002	0.088	0.003

CFI, comparative fit index; △CFI, the change in comparative fit index; RMSEA, root mean square error of approximation; △RMSEA, the change in root mean square error of approximation.

## 4. Discussion

In this study, we found that the Chinese version of the TGNS demonstrates a satisfactory measure of validity and reliability for assessing Chinese college students.

The item analysis revealed a high level of differentiation among the items in the Chinese version of the TGNS, with strong correlation between the items and the overall scale. This finding provides a foundational confirmation that the Chinese version of the TGNS is reasonably accurate. Moreover, the scale demonstrated excellent internal consistency with an overall coefficient of 0.947. This indicates that the Chinese version of the TGNS exhibits a similarly high level of internal stability as observed in the original scale.

Exploratory factor analysis and confirmatory factor analysis both showed that the structure of the Chinese version of the TGNS was consistent with the original single-factor structure of the TGNS, with all model fit indices falling within an acceptable range. These results suggested that the Chinese version of the TGNS exhibits good construct validity and meets psychometric criteria. While Tam (2022) previously posited that gratitude should inherently encompass four distinct dimensions (gratitude intensity facet, gratitude frequency facet, gratitude span facet, and gratitude expression facet), the results obtained from both the original scale and the Chinese version of the TGNS unequivocally indicate support for a one-dimensional structure. This finding suggests that the primary focus of the TGNS lies in measuring the shared emotional essence rather than individual variations in emotional display. Consequently, this result effectively clarifies why the structure of the TGNS maintains cross-cultural consistency.

In terms of criterion validity, the current study employed the Gratitude Questionnaire-6, the Pro-Environment Behavior Scale, and the Epidemic Center Depression Scale as measures.

Consistent with expectations, trait gratitude to nature is moderately correlated with trait gratitude. This association could be attributed to the heightened sensitivity toward benefits that individuals with higher trait gratitude possess, regardless of whether these benefits stem from humans or the natural world (Berger et al., 2019; Jacobs, 2022; Tam, 2022). However, the TGNS offers a more precise and differentiated assessment of an individual’s tendency to express gratitude toward the natural world. This particular aspect cannot be directly captured through the use of a general trait gratitude scale, such as GQ-6.

Additionally, consistent with previous studies (Kearns, 2020; Tam, 2022), the results of this study indicated that trait gratitude to nature is positively correlated with pro-environmental behavior. A possible explanation for this finding is provided by the amplifying the good theory of gratitude, which posits that gratitude acts as a reinforcer (Watkins, 2013), enhancing the magnitude of benefit signaling. Therefore, individuals with greater gratitude to nature may exhibit increased awareness of the benefits received from the environment, leading to a greater desire to reciprocate, manifesting through intentional behaviors. Nevertheless, it is essential to emphasize that the correlation observed in this study between trait gratitude to nature and pro-environmental behavior, although statistically significant, is not particularly strong. This suggests that there may be additional influencing factors at play between gratitude for nature and pro-environmental behavior, such as a sense of indebtedness. Tam (2022) conducted a study revealing that participants in the gratitude to nature condition experienced both a higher level of gratitude toward nature and feelings of indebtedness toward nature. This finding indicates that acknowledging and benefiting from nature not only generates gratitude to nature but may also invokes a sense of indebtedness. While the former encourages individuals to engage in pro-environmental behavior, the latter may hinder the implementation of reciprocal actions, such as pro-environmental behavior, due to the pressures of repayment (Harari et al., 2022). Consequently, further investigation is necessary to obtain a more comprehensive understanding of these complex dynamics.

Moreover, we have found that trait gratitude to nature was negatively associated with depression. According to the cognitive focus shift hypothesis of gratitude, gratitude has the ability to shift one’s thinking and action tendencies from self-focus to other-focus modes (Watkins, 2013; Liang, 2022). Gratitude to nature reinforces this effect by promoting recognition and appreciation of the well-being and goodwill that nature provides. By including nature within oneself (Jacobs, 2022), gratitude to nature can further strengthen the sense of attachment to nature and cultivate a belief in the mutual benefits of pro-environmental behaviors. Consequently, gratitude to nature may help prevent negative self-focused thought patterns such as brooding rumination, ultimately reducing depression.

Lastly, we performed a gender invariance assessment on the Chinese version of the TGNS, and the findings confirm that it exhibits complete gender invariance. Generally, women tend to exhibit a higher level of gratitude compared to men (Froh et al., 2009). Therefore, in order to minimize measurement biases arising from gender variations, it is crucial to examine the cross-gender equivalence of gratitude scales. The Chinese version of the TGNS has been validated as an effective tool for assessing participants’ level of trait gratitude to nature, independent of gender differences.

Based on the findings, this study has two additional strengths. Firstly, the Chinese version of the TGNS serves as a useful tool for researchers interested in studying the relationship between gratitude to nature and pro-environmental behavior within the context of Chinese culture. To our knowledge, this study is the first to examine the applicability of the Chinese version of the TGNS in Chinese mainland. The findings of this study not only provide valuable insights into the level of gratitude to nature among university students in Chinese mainland but also serve as a foundational dataset for gratitude educators to develop intervention strategies aimed at enhancing pro-environmental behavior. Secondly, our study has directly confirmed the inverse association between gratitude to nature and depression. Previous research has consistently demonstrated a positive correlation between the ability of gratitude toward nature and engagement in nature walks, which in turn has been linked to a reduction in the likelihood of experiencing depression (Bratman et al., 2019; Büssing et al., 2021). As a result, the findings of this study open up potential avenues for developing gratitude to nature interventions aimed at mitigating depression.

While this study has made some contributions, it is crucial to acknowledge its limitations. Firstly, it is essential to acknowledge the limitation of this study, which focused solely on university students, thus neglecting individuals from various occupational backgrounds and diverse age groups. To address this limitation and strengthen the validity of the conclusions, future research should include participants from a broader spectrum of professions and age ranges. This would help provide more comprehensive validation of the Chinese version of the TGNS. Secondly, the primary method employed in this study was self-reporting, which introduces a certain degree of subjectivity in the assessment of variable relationships. To mitigate this limitation, future research could consider incorporating more explicit or objective evaluation indicators. For example, incorporating behavioral response indicators, such as donating to the Nature Conservancy and adopting various low-carbon lifestyle habits, can be considered to improve the selection of evaluation criteria. Thirdly, the relationships between variables in this study were derived using a cross-sectional approach, which does not establish causal direction. To further enhance our understanding, future research can employ a longitudinal tracking approach. This would allow for a more refined examination of specific variable predictive relationships, such as the connection between trait gratitude to nature and pro-environmental behavior, as well as depression.

## 5. Conclusion

In summary, this study has confirmed that the Chinese version of the TGNS is a reliable and valid measure. The results showed that the scale has good internal consistency, criterion validity, construct validity, test–retest reliability, and gender invariance. Consequently, the Chinese version of the TGNS could effectively assess the trait gratitude to nature in Chinese university students. We hope that our findings would encourage future research to employ this scale when investigating similar areas and that it will be of use to practitioners in their work.

## Data availability statement

The original contributions presented in the study are included in the article/Supplementary material, further inquiries can be directed to the corresponding author.

## Ethics statement

The studies involving human participants were reviewed and approved by the Institutional Review Board of the Inner Mongolia Normal University. The participants provided their written informed consent to participate in this study.

## Author contributions

XL, HL, and TJ contributed to the conception and design of the study and wrote sections of the manuscript. XL organized the database and performed the statistical analysis. XL, JZ, YA, MH, and YW participated in the data collection. XL and HL wrote the first draft of the manuscript. All authors contributed to the manuscript revision, read, and approved the submitted version.

## Funding

This study was supported by the National Social Science Fund of China (22XSH002), the Inner Mongolia Natural Science Foundation project (2021LHBS03004), the Inner Mongolia Philosophy and Social Science Planning Project (2019NDC100), the Fundamental Research Funds for the Inner Mongolia Normal University (2022JBQN115), the High-level Talents Research Grant of Inner Mongolia Normal University (2019YJRC016), and the Inner Mongolia Normal University Graduate Scientific Research and Innovation Fund Project (CXJJS22011).

## Conflict of interest

The authors declare that the research was conducted in the absence of any commercial or financial relationships that could be construed as a potential conflict of interest.

## Publisher’s note

All claims expressed in this article are solely those of the authors and do not necessarily represent those of their affiliated organizations, or those of the publisher, the editors and the reviewers. Any product that may be evaluated in this article, or claim that may be made by its manufacturer, is not guaranteed or endorsed by the publisher.
